# Phenotypic and Genotypic Assays to Evaluate Coagulase-Negative Staphylococci Biofilm Production in Bloodstream Infections

**DOI:** 10.3390/microorganisms12010126

**Published:** 2024-01-08

**Authors:** Giulia Grassia, Jessica Bagnarino, Mariangela Siciliano, Daniela Barbarini, Marta Corbella, Patrizia Cambieri, Fausto Baldanti, Vincenzina Monzillo

**Affiliations:** 1 Microbiology and Virology Department, Fondazione IRCCS Policlinico San Matteo, 27100 Pavia, Italy; g.grassia@smatteo.pv.it (G.G.); d.barbarini@smatteo.pv.it (D.B.); m.corbella@smatteo.pv.it (M.C.); p.cambieri@smatteo.pv.it (P.C.); f.baldanti@smatteo.pv.it (F.B.); vincenzina.monzillo@unipv.it (V.M.); 2 Cell Factory, Fondazione IRCCS Policlinico San Matteo, 27100 Pavia, Italy; m.siciliano@smatteo.pv.it; 3 Department of Clinical, Surgical, Diagnostics and Pediatric Sciences, University of Pavia, 27100 Pavia, Italy; 4 Department of Internal Medicine and Medical Therapy, Faculty of Medicine, University of Pavia, 27100 Pavia, Italy

**Keywords:** biofilm, bloodstream infection, catheter, coagulase-negative staphylococci (CoNS), *S. epidermidis*

## Abstract

Coagulase-negative staphylococci (CoNS) are commensal on human body surfaces and, for years, they were not considered a cause of bloodstream infection and were often regarded as contamination. However, the involvement of CoNS in nosocomial infection is increasingly being recognized. The insertion of cannulas and intravascular catheters represents the primary source of CoNS entry into the bloodstream, causing bacteremia and sepsis. They owe their pathogenic role to their ability to produce biofilms on surfaces, such as medical devices. In this study, we evaluate the adhesive capacity of CoNS isolated from blood cultures by comparing a spectrophotometric phenotypic assay with genotypic analysis based on the evidence of the *ica* operon. We retrospectively reviewed the database of CoNS isolated from blood cultures from January to December 2021 that were considered responsible for 361 bloodstream infections. Eighty-nine CoNS were selected among these. Our data show that *Staphylococcus epidermidis* was the predominant species isolated, expressing greater adhesive capacities, especially those with the complete operon. Knowledge of the adhesive capabilities of a microorganism responsible for sepsis can be useful in implementing appropriate corrective and preventive measures, since conventional antibiotic therapy cannot effectively eradicate biofilms.

## 1. Introduction

The coronavirus disease 2019 (COVID-19) pandemic has had a considerable impact on healthcare, especially for a large number of patients requiring intensive care. The management of patients became more difficult, from diagnostic to safety measures, including delays in treatment delivery [1]. Modifications in standards of care resulted in a growing number of coagulase-negative staphylococci (CoNS) isolated from blood cultures, which remains an urgent problem to be solved. Numerous studies have shown a significant increase in incidence, especially in intensive care units, during this critical period [2].

The genus *Staphylococcus* includes Gram-positive cocci that usually grow in a cluster-like arrangement. Staphylococci belong to the *Staphylococcaceae* family; most have a diameter of 0.5–1.5 μm and are immobile. They are aerobic or facultative anaerobic, non-spore-forming bacteria that are usually non-capsulated or present with the formation of a very limited capsule. Staphylococci differ mainly in their ability to coagulate blood by producing the coagulase enzyme and in their ability to ferment mannitol. *Staphylococcus aureus*, in fact, produces coagulase and ferments mannitol, while CoNS do not produce coagulase and can only rarely ferment mannitol [3]. Staphylococci are ubiquitous and frequently colonize the skin and mucous membranes of humans; in particular, they can also be isolated from the oral cavity and mammary and intestinal glands, as well as from the urogenital tract and the upper respiratory tract [4]. There is strong evidence of CoNS involvement in severe diseases: osteomyelitis, otitis, wound infections, endophthalmitis, urinary tract infections, meningitis, or even pneumonia caused by *S. epidermidis*, *Staphylococcus saprophyticus*, *Staphylococcus lugdunensis*, and *Staphylococcus schleiferi* [5,6]. When colonizing staphylococci gain entry into the bloodstream, they can potentially lead to a systemic infection. The pathogenic role of CoNS, however, is attributed to their ability to anchor themselves to surfaces and remain adherent, forming a biofilm, which, therefore, represents the best virulence factor of this species [4,7].

Biofilm consists of multilayered cell clusters embedded in a matrix of extracellular polysaccharide that facilitate the adherence of microorganisms. Bacteria can attach to a wide range of surfaces, the physicochemical properties of which influence the adhesion behaviour of cells on the surface of the material and the subsequent process of biofilm formation [8]. Charge and hydrophobicity are two major components that influence bacterial–surface interactions. The chemical properties of the bacterial cell envelope may vary, but as a general rule, they are often negatively charged; therefore, surfaces with positive or neutral charges are more easily colonized than those with negative charges. Hydrophobic interactions can also be established between microorganisms and devices with hydrophobic surfaces [9]. The phenomenon of adhesion depends not only on the factors of the microorganism, but also on the characteristics of the material, such as the roughness, surface composition, topography and functional chemical group modifications of the material surface. The surface modification of biomaterials is a potential strategy to prevent bacteria from attaching and forming biofilms [8]. In the clinical field, an effective modification could be coating with antibiotics [10]. The insertion of cannulas and intravascular catheters (for the administration of liquids or drugs, hemodynamic monitoring, hemodialysis, etc.) represents the first source of CoNS entry into the bloodstream [6]. One of the major complications is represented by the possible establishment of catheter-related sepsis, due to the biofilm formation and subsequent dissemination of microrganisms. The mechanism by which CoNS attach to prosthetic material and elaborate what is now termed a biofilm is being increasingly better understood. One important element in this process is the *ica* operon, a gene cluster encoding the production of polysaccharide intercellular adhesin (PIA) [11]. The chromosomal *ica* gene locus comprises four intercellular adhesion genes (*icaA*, *icaB*, *icaC*, and *icaD*) and one regulator gene (*icaR*), which seems to function as a repressor [12]. Some in vitro tests using UDP-N-acetylglucosamine as a substrate have shown that the transmembrane glycotransferase *icaA* is the enzyme most involved in the production of PIA. However, it has also been demonstrated that the transferase activity of *icaA* is enhanced through the simultaneous presence of *icaD*. *IcaA* and *IcaD* are found in the plasma membrane, and the former is a 412 amino acid polypeptide with four transmembrane domains, while *IcaD* is much smaller, with only 101 amino acids with two potential transmembrane domains. The *icaAD* complex has been shown to produce a PIA with a maximum length of only 20 amino acid residues, while the co-expression of the transmembrane protein *IcaC* is required for further elongation. Only recently has it been discovered that *IcaC* is a member of the membrane-bound acyltransferases and presumably responsible for the succinylation of PIA [13]. *IcaB*, on the other hand, is a secreted protein composed of 259 amino acids, responsible for the de-N-acetylation of PIA and essential for biofilm formation and virulence in *S. epidermidis*, as it introduces a net positive charge in PIA, which causes the bacterial cell to adhere stably to the surfaces of the devices [14]. The transcription of the *icaADBC* operon is regulated by a repressor, called *icaR*, positioned upstream of the operon, and belonging to the *TetR* family of transcriptional regulators. It binds to a specific DNA region upstream of *icaA*, resulting in the strong suppression of *icaADBC* transcription. Consequently, the deletion of *icaR* leads to overproduction of PIA. Aminoglycoside antibiotics can also induce biofilm formation by interfering with the binding of *icaR* to DNA [15]. In staphylococci, the adhesion to the surface of catheters is also mediated by covalently membrane-anchored proteins like cell-wall-anchored (CWA) proteins [16], noncovalently associated proteins (extracellular glycopolymer WTAs) [13], and other mucopolysaccharide factors (wall teichoic acids and lipoteichoic acids and PIA) [16]. The precise percentage of how many positive blood cultures are due to contamination varies considerably among the many studies that have attempted to estimate that number. Approximately 30% to 40% of nosocomial bloodstream infections are caused by CoNS; nevertheless, there is agreement that *S. epidermidis* is among the most frequent bacterial sources underlying bacteremia and sepsis, with a 90% prevalence [13,17]. In this study, we evaluate the adhesive capacity of CoNS isolated from blood cultures by comparing a spectrophotometric phenotypic assay with genotypic analysis based on the evidence of the *ica* operon.

## 2. Materials and Methods

### 2.1. Study Design

The study was conducted by the Fondazione IRCCS Policlinico San Matteo Pavia, Italy. We retrospectively reviewed 89 CoNS isolated from blood cultures from January to December 2021 that were responsible for bloodstream infections. They were stratified as follows: 56 *Staphylococcus epidermidis*, 13 *Staphylococcus hominis*, 13 *Staphylococcus haemolyticus*, 5 *Staphylococcus capitis*, and 2 *Staphylococcus lugdunensis*.

### 2.2. Bacterial Isolates 

The bacterial strains were stored in preservation media (BIO-RAD©, Hercules, CA, USA) at room temperature and revitalized for the purpose of the study. A small amount of sterile 0.9% saline solution (NaCl) was added to each tube to resuspend the microorganism from the agarized medium, after which a drop was transferred to a Columbia Agar with 5% Horse Blood (bioMérieux, St. Louis, MO, USA) plate using a Pasteur pipette. After isolation, the plates were incubated at 37 °C for 24 h.

### 2.3. Phenotypic Assay 

Biofilm production was investigated using Christensen’s spectrophotometric method, which allows quantitative evaluation of the phenomenon [18]. 

The strains were resuspended in 10 mL of Tryptic Soy Broth (TSB) (Becton Dickinson, Franklin Lakes, NJ, USA) supplemented with 0.25% *w*/*v* casamino acid and glucose (Sigma-Aldrich, St. Louis, MO, USA). After 24 h incubation at 37 °C, dilutions (1:1000) were performed in TSB additioned as described above to have a bacterial suspension of about 10^5^ CFU/mL. Of each dilution, 200 µL was deposited in triplicate in the wells of a flat-bottom microtiter plate (Greiner BIO-ONE©, Kremsmünster, Austria) that was incubated at 37 °C for 18 h. The culture broth was removed from each well and washed twice with sterile water, and the microorganisms were fixed with absolute alcohol (Sigma-Aldrich), after which they were washed once more and stained with crystal violet (Sigma-Aldrich) for 10 min. The dye was removed from each well, two more washes were performed, and the plate was allowed to dry at room temperature.

After the BIO-RAD© (USA) instrument calibration, the spectrophotometric reading was taken at a wavelength of 570 nm, and the mathematical average of the 3 optical density (OD) values relative to each strain was calculated. The observed OD value was proportional to the biofilm production of the microorganisms and allowed us to classify them according to Christensen’s scheme into excellent producers when OD > 0.240, weak producers when OD was between 0.120 and 0.240, and non-producers when OD < 0.120 (Christensen et al., 1982) [18].

### 2.4. Genotypic Assays 

#### 2.4.1. DNA Extraction 

DNA was extracted using the DNeasy^®^ UltraClean^®^ Microbial Kit (Qiagen, Hilden, Germany); cells were resuspended in a bead solution and added to a bead-beating tube containing beads. Then, lysis solution was added. The microorganisms were lysed via a combination of heat, detergent, and mechanical force against specialized beads. The cellular components were lysed via mechanical action using a specially designed Vortex Adapter on a standard vortex. The DNA released from the lysed cells was bound to a silica spin filter. The spin filter was washed, and the DNA was recovered in a DNA-free Tris buffer.

#### 2.4.2. PCR Assays

Genotypic assay was conducted via polymerase chain reaction (PCR) (Applied Biosystems™ MiniAmp™ Thermal Cycler, Thermo Fisher Inc., Waltham, MA, USA) and amplification of the four genes of the *icaADBC* operon, responsible for PIA production, with specific primers [12] (Table 1). 

Amplification mixes contained buffer 10× with MgCl_2_ 15 mM, MgCl_2_ (25 mM), Taq polymerase (5U/μL), dNTPs (10 mM), primers (10 μM), and 2 μL of genomic DNA. PCR conditions comprised an initial 5 min denaturation step at 95 °C, followed by 35 cycles of 94 °C for 30 s, and 58 °C for 30 s for *icaC*, while for *icaA*, *icaD*, and *icaB*, this phase was set to 55 °C for 30 s and extended for 2 min at 72 °C. Amplified products were visualized via electrophoresis on 2% agarose gels stained with ethidium bromide. 

### 2.5. Statistical Analysis

A descriptive analysis was performed for the distribution of the genes of interest, as well as biofilm production measured via the OD. Moreover, a non-parametric test was calculated using Kruskal–Wallis for comparison between each genotype and categories, with a *p* < 0.0001 that showed significant differences among categories. All analysis were carried out with MedCalc statistical software Version 22.017.

## 3. Results

**Figure 1:**
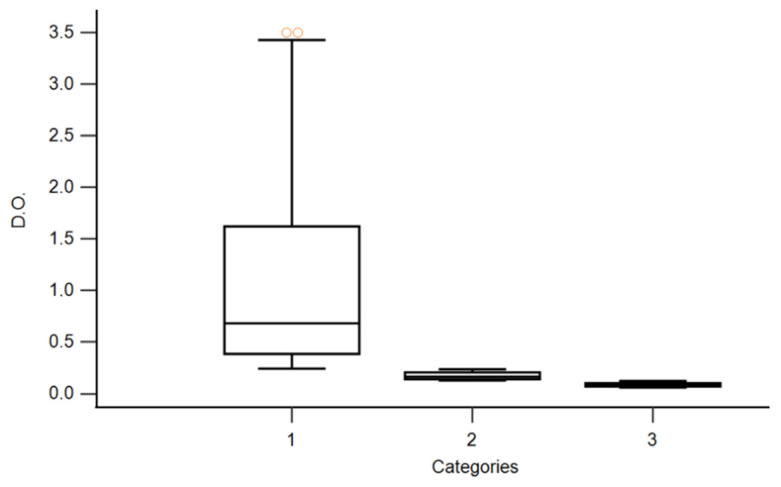
Box plot of distribution of the optical density (OD) of CoNS grouped into three categories (excellent producers k = 1, weak producers k = 2, no producers k = 3). The Kruskal–Wallis test showed significant differences among categories (*p* < 0.0001). The circles above the top bar indicate the outliers, i.e., the values that deviate from the case history.

We retrospectively reviewed the database of CoNS isolated from blood cultures between January 2021 and December 2021. In our results, Staphylococci were considered to be responsible for 490 bloodstream infectious episodes, of which 361/490 (73.7%) were sustained due to CoNS. Among them, we randomly selected 89 CoNS for our analysis. The OD measured on microplates showed a wide distribution of values, ranging from a minimum of 0.06 to a maximum of 3.5. According to criteria established by Christensen, 49/89 (55%) were excellent producers, 20/89 (22.5%) were weak producers, and 20/89 (22.5%) were non-producers (Figure 1).

The genotypic assay showed that 42/89 (47.2%) presented the complete *ica* operon, 44/89 (49.4%) did not have the operon, 3/89 strains (3.4%), otherwise, presented one or two genes of the operon. The percentages of strains divided into the three categories associated with the presence of the complete operon, range, medians, and CI 95% are reported in Table 2. The chi-squared test showed significant differences among categories (*p* < 0.05).

As regards *S. epidermidis*, 42 of 57 (73.6%) strains had the complete *ica* operon, of which 33 (78.6%) were excellent biofilm producers, 4 (9.5%) were weak producers, and 5 (11.9%) were non-producers. The *S. epidermidis* strain that only tested positive for the *icaA* and *icaC* genes was found to be a weak producer. Comparing, instead, the fourteen strains of *S. epidermidis* lacking all four genes, it can be said that six (42.85%) were excellent producers, five (35.71%) were weak producers, and three (21.42%) were non-producers. The relationship between the optical density and the presence or not of the *ica* operon is represented in Table 3.

## 4. Discussion

The CoNS, including *S. epidermidis*, are ubiquitous in nature, residing on the skin of healthy individuals as normal flora. The CoNS are among the most commonly isolated microorganisms from blood samples. Compared to *Staphylococcus aureus* strains, which are classified as invasive pathogens, the clinical significance of CoNS needs to be proven. In fact, it is essential to estimate whether their presence represents true bacteremia or sample contamination. Several studies have shown that CoNS can cause serious bloodstream infections [21,22,23]. These bacterial species have emerged as common nosocomial pathogens with the ability to form biofilms on biotic, as well as abiotic, surfaces. This ability has led to their involvement in persistent human colonization and infections giving rise to serious health problems. Given that biofilm is an important virulence factor that is associated with antibiotic resistance for these pathogens, early detection in clinical specimen would have a significant impact on the management of staphylococcal nosocomial infections [19].

Our data show that CoNS, during the period of the COVID-19 pandemic, were considered responsible for an increased number of episodes of bloodstream infections. Taking this into consideration, the study was carried out to investigate the production of biofilms by CoNS isolated from blood cultures between January 2021 and December 2021, in different departments of IRCCS Policlinico San Matteo Foundation.

Among 89 CoNS samples, *S. epidermidis* was the most common isolate, accounting for more than half (n = 56, 62.9%) of the total, followed by *S. hominis* (n = 13, 14.6%), *S. haemolyticus* (n = 13, 14.6%), *S. capitis* (n = 5, 6%), and *S. lugdunensis* (n = 2, 2.2%) (Table 4). Regarding the distribution of isolates in the different departments of our hospital, it can be observed that the highest number of CoNS (49/89) was found in the General Intensive Care Unit (ICU) (Table 4). This finding confirms what has already been reported in the literature; namely, that long hospital stays and the use of intravascular devices are more prone to colonization of the catheter surface and, consequently, to bloodstream infections [24].

A phenotypic evaluation of biofilm production was conducted on the 89 CoNS strains using the Christensen spectrophotometric method. From the study, we can observe that 49/89 (55.06%) of the strains examined were found to be excellent biofilm producers, 20/89 (22.47%) were weak producers, and 20/89 (22.47%) were found to be non-biofilm-producers. In the category of excellent producers, the range was 3.50–0.24, the median of which was 0.68. In the category of weak producers, the range was 0.23–0.13, with a median was 0.17. In the non-producer category, the range was 0.12–0.06, the median of which was 0.09.

An interesting finding of this study is that only *S. epidermidis* presented the entire operon, in particular, 42 of 57 strains (73.7%), of which 36 (85.1%) were biofilm producers and 5 (11.9%) were non-producers. Only one *S. epidermidis* strain tested positive for the *icaA* and *icaC* genes and was found to be a weak producer. This could be due to various factors such as the environment, nutrition, subinhibitory concentration of certain antibiotics, and stress (temperature, osmolarity) which might play a significant role in biofilm formation, resulting in the varied frequency of biofilm producers among clinical isolates [25,26].

Instead, considering the 14/57 (24.5%) strains of *S. epidermidis* negative to the genotypic test for all four genes, it can be said that three (21.42%) were non-producers, while 11/14 (78.6%) were biofilm producers. This discrepancy between the phenotypic and genotypic tests is explained by the multifactorial genesis of the biofilm used by Staphylococci, and it is in accordance with data from the literature [12]. As previously mentioned, the biosynthetic genes responsible for the production of PIA are located in an operon called *icaADBC*, which is highly prevalent among invasive CoNS isolates, explaining why it has been proposed as a marker to discriminate contaminating strains from invasive ones [27,28]. The production of PIA is related to the pathogenesis of these microorganisms; however, its presence alone is not sufficient for the formation of a biofilm, which seems to be dependent on environmental conditions and cell concentration. This demonstrates how it represents a key role in the survival of the microorganism, providing it with a selective advantage in the etiology of subsequent infections [29].

Our study could be expanded by considering various blood-culture-sampling sites and various implantable medical devices. A future perspective could be to study other factors that influence biofilm formation.

The eradication of biofilms from medical devices represents a major challenge for researchers and clinicians today, as antibiotic-based treatments often fail to completely suppress the infection, despite high dosages and long-term treatment [30].

Considering the various infections caused by medical devices, there are many studies in the literature explaining methods of preventing and treating biomaterial-related biofilm infection: antibacterial coatings and the surface modification of biomaterials.

Antibacterial coatings depend on the covalent immobilization of antimicrobial agents on the coating surface and drug release to prevent and combat infection, while the surface modification of biomaterials affects the adhesion behavior of cells on the surfaces of implants and the subsequent biofilm formation process by altering the physical and chemical properties of the implant material surface [8].

One of the latest methods tested includes the use of nanoparticles like silver nanoparticles (AgNPs) as alternative antimicrobial agents [31]. The multiple biofilm-forming mechanisms make it highly difficult to prevent CoNS infection once it gains access during device insertion. Therefore, multitargeted antimicrobials such as AgNPs could provide effective barriers to prevent colonization and biofilm formation by CoNS.

## 5. Conclusions

The results of this study show that most CoNS strains are capable of developing biofilms, especially those with the complete *ica* operon, thus increasing the pathogenicity of these strains, which are believed to be responsible for catheter colonization, bacteremia, and sepsis. This approach allowed us to underline an increase in the incidences of bloodstream infections supported by CoNS biofilm producers, especially in patients with temporary or permanent biomedical devices. However, our study confirms what has been reported previously: that the *ica* operon is largely involved in biofilm production, although it is not the only contributing factor. In fact, some strains have been shown to be capable of producing biofilms, despite lacking this operon, because the genesis of biofilms is considered multifactorial.

Knowing the adhesive capabilities of a micro-organism responsible for sepsis can be useful in implementing appropriate corrective and preventive measures, such as the use of devices with antibacterial coatings, as conventional antibiotic therapy cannot effectively eradicate biofilms. The use of antibacterial coatings in medical materials not only prevents bacterial adhesion but also directly fights biofilm infections by releasing antibacterial drugs.

In conclusion, to better solve the problem of biofilm resistance, further in-depth research is needed, with the development of new antibacterial coatings (biodegradable and biocompatible) and the improvement of prevention by optimizing medical materials and hospital infection control measures.

## Figures and Tables

**Table 1:** Primers and their sequences used in the study.

Gene Target	Sequences	bp	Reference
***icaA***	F5′-TCTCTTGCAGGAGCAATCAA	188	[19]
	R5′-TCAGGCACTAACATCCAGCA		
***icaB***	F5′-ATGGCTTAAAGCACACGACGC	526	[20]
	R5′-TATCGGCATCTGGTGTGACAG		
***icaC***	F5′-ATCATCGTGACACACTTACTAACG	934	[12]
	R5′-CTCTCTTAACATCATTCCGACGCC		
***icaD***	F5′-ATGGTCAAGCCCAGACAGAG	198	[19]
	R5′-CGTGTTTTCAACATTTAATGCAA		

**Table 2:** Summary of the results.

Phenotypic Assay	Frequency	Range	Median (OD)	95% CI for the Median	Complete Operon
Excellent producer	49/89 (55.0%)	0.240–3.50	0.68	0.46–1.21	33/49 (67.3%)
Weak producer	20/89 (22.5%)	0.130–0.230	0.17	0.14–0.20	4/20 (20.0%)
No producer	20/89 (22.5%)	0.06–0.120	0.09	0.1–0.1	5/20 (25.0%)

**Table 3:** Correlation between the biofilm production with the optical densities (phenotypic assay) and the presence of the *ica* operon (genotypic assay).

Samples		Phenotypic Assay		Genotypic Assay			
Strain	Species	Average o.d.	Interpretation	*icaA*	*icaD*	*icaB*	*icaC*
30,678	*S. epidermidis*	3.50	EP	+	+	+	+
29,789	*S. epidermidis*	3.50	EP	+	+	+	+
29,216	*S. epidermidis*	3.43	EP	+	+	+	+
30,667	*S. epidermidis*	3.33	EP	+	+	+	+
30,203	*S. epidermidis*	3.30	EP	+	+	+	+
30,383	*S. epidermidis*	2.37	EP	+	+	+	+
30,428	*S. epidermidis*	2.25	EP	+	+	+	+
30,371	*S. epidermidis*	2.24	EP	+	+	+	+
30,344	*S. epidermidis*	1.94	EP	+	+	+	+
30,710	*S. epidermidis*	1.78	EP	+	+	+	+
29,533	*S. epidermidis*	1.74	EP	+	+	+	+
30,385	*S. epidermidis*	1.65	EP	+	+	+	+
30,575	*S. epidermidis*	1.61	EP	+	+	+	+
29,383	*S. epidermidis*	1.59	EP	+	+	+	+
30,164	*S. epidermidis*	1.59	EP	-	-	-	-
29,317	*S. epidermidis*	1.52	EP	+	+	+	+
30,677	*S. epidermidis*	1.37	EP	+	+	+	+
30,077	*S. epidermidis*	1.23	EP	+	+	+	+
29,981	*S. epidermidis*	1.14	EP	+	+	+	+
30,455	*S. epidermidis*	1.02	EP	-	-	-	-
30,697	*S. epidermidis*	0.86	EP	+	+	+	+
29,581	*S. epidermidis*	0.82	EP	+	+	+	+
30,338	*S. epidermidis*	0.77	EP	+	+	+	+
29,412	*S. epidermidis*	0.73	EP	+	+	+	+
30,306	*S. epidermidis*	0.68	EP	+	+	+	+
30,740	*S. epidermidis*	0.67	EP	+	+	+	+
29,525	*S. epidermidis*	0.66	EP	-	-	-	-
30,440	*S. lugdunensis*	0.64	EP	-	-	-	-
30,418	*S. epidermidis*	0.63	EP	-	-	-	-
30,064	*S. epidermidis*	0.62	EP	+	+	+	+
29,954	*S. hominis*	0.51	EP	-	-	-	-
29,638	*S. hominis*	0.46	EP	-	-	-	-
29,993	*S. epidermidis*	0.43	EP	+	+	+	+
29,668	*S. epidermidis*	0.43	EP	+	+	+	+
29,743	*S. hominis*	0.43	EP	-	-	-	-
30,789	*S. epidermidis*	0.42	EP	+	+	+	+
30,607	*S. capitis*	0.39	EP	-	-	-	-
30,702	*S. hominis*	0.36	EP	+	+	-	-
30,478	*S. epidermidis*	0.34	EP	+	+	+	+
29,769	*S. hominis*	0.32	EP	-	-	-	-
29,540	*S. epidermidis*	0.29	EP	+	+	+	+
30,735	*S. haemolyticus*	0.28	EP	-	-	-	-
29,798	*S. epidermidis*	0.27	EP	-	-	-	-
29,726	*S. epidermidis*	0.27	EP	+	+	+	+
30,706	*S. epidermidis*	0.26	EP	+	+	+	+
30,530	*S. haemolyticus*	0.26	EP	-	-	-	-
30,242	*S. hominis*	0.25	EP	-	-	-	-
30,595	*S. epidermidis*	0.25	EP	+	+	+	+
30,239	*S. epidermidis*	0.24	EP	-	-	-	-
29,409	*S. epidermidis*	0.23	WP	+	+	+	+
30,359	*S. capitis*	0.22	WP	-	-	-	-
29,846	*S. epidermidis*	0.21	WP	-	-	-	-
29,655	*S. hominis*	0.21	WP	-	-	-	-
29,808	*S. epidermidis*	0.21	WP	+	+	+	+
28,995	*S. haemolyticus*	0.20	WP	-	-	-	-
30,244	*S. epidermidis*	0.20	WP	-	-	-	-
30,296	*S. haemolyticus*	0.18	WP	-	-	-	-
30,288	*S. hominis*	0.18	WP	-	-	-	-
30,417	*S. hominis*	0.17	WP	+	-	-	-
29,618	*S. epidermidis*	0.16	WP	-	-	-	-
29,972	*S. epidermidis*	0.15	WP	+	+	+	+
30,358	*S. haemolyticus*	0.15	WP	-	-	-	-
30,291	*S. epidermidis*	0.15	WP	-	-	-	-
30,319	*S. epidermidis*	0.14	WP	-	-	-	-
30,013	*S. hominis*	0.14	WP	-	-	-	-
30,773	*S. epidermidis*	0.13	WP	+	+	+	+
30,387	*S. haemolyticus*	0.13	WP	-	-	-	-
29,657	*S. epidermidis*	0.13	WP	+	-	-	+
29,378	*S. hominis*	0.13	WP	-	-	-	-
30,218	*S. epidermidis*	0.12	NP	-	-	-	-
29,852	*S. haemolyticus*	0.12	NP	-	-	-	-
30,539	*S. haemolyticus*	0.11	NP	-	-	-	-
29,797	*S. haemolyticus*	0.11	NP	-	-	-	-
30,814	*S. epidermidis*	0.10	NP	-	-	-	-
30,807	*S. capitis*	0.10	NP	-	-	-	-
29,834	*S. haemolyticus*	0.10	NP	-	-	-	-
29,313	*S. epidermidis*	0.10	NP	+	+	+	+
29,297	*S. epidermidis*	0.09	NP	+	+	+	+
29,205	*S. epidermidis*	0.09	NP	-	-	-	-
29,416	*S. epidermidis*	0.08	NP	+	+	+	+
29,522	*S. epidermidis*	0.08	NP	+	+	+	+
29,934	*S. capitis*	0.08	NP	-	-	-	-
29,363	*S. epidermidis*	0.07	NP	+	+	+	+
29,440	*S. hominis*	0.07	NP	-	-	-	-
29,147	*S. hominis*	0.07	NP	-	-	-	-
29,367	*S. haemolyticus*	0.07	NP	-	-	-	-
30,761	*S. capitis*	0.06	NP	-	-	-	-
30,318	*S. haemolyticus*	0.06	NP	-	-	-	-
29,059	*S. haemolyticus*	0.06	NP	-	-	-	-

EP, excellent producer; WP, weak producer; NP, non-producer.

**Table 4:** CoNS strains isolated in each department.

Department	Total	*S. epidermidis*	*S. hominis*	*S. haemolyticus*	*S. capitis*	*S. lugdunensis*
General ICU	49	36	9	4		
Surgeries	5	4			1	
Pulmonology	5	2	2	1		
Hematology	10	3		5		2
Oncologies	9	6	1	2		
Cardiology	8	5	1	1	1	
Neonatal intensive care unit	3				3	
Total	89	56	13	13	5	2

## Data Availability

Data are contained within the article.
